# Correction to “FLOT1 promotes tumor development, induces epithelial–mesenchymal transition, and modulates the cell cycle by regulating the Erk/Akt signaling pathway in lung adenocarcinoma”

**DOI:** 10.1111/1759-7714.14990

**Published:** 2023-06-05

Zhang L, Mao Y, Mao Q, Fan W, Xu L, Chen Y, et al. FLOT1 promotes tumor development, induces epithelial–mesenchymal transition, and modulates the cell cycle by regulating the Erk/Akt signaling pathway in lung adenocarcinoma. *Thorac Cancer* 2019;**10**(4): 909–17. 10.1111/1759‐7714.13027

The authors would like to amend the images in Figure 2a. The correct figure is shown below:

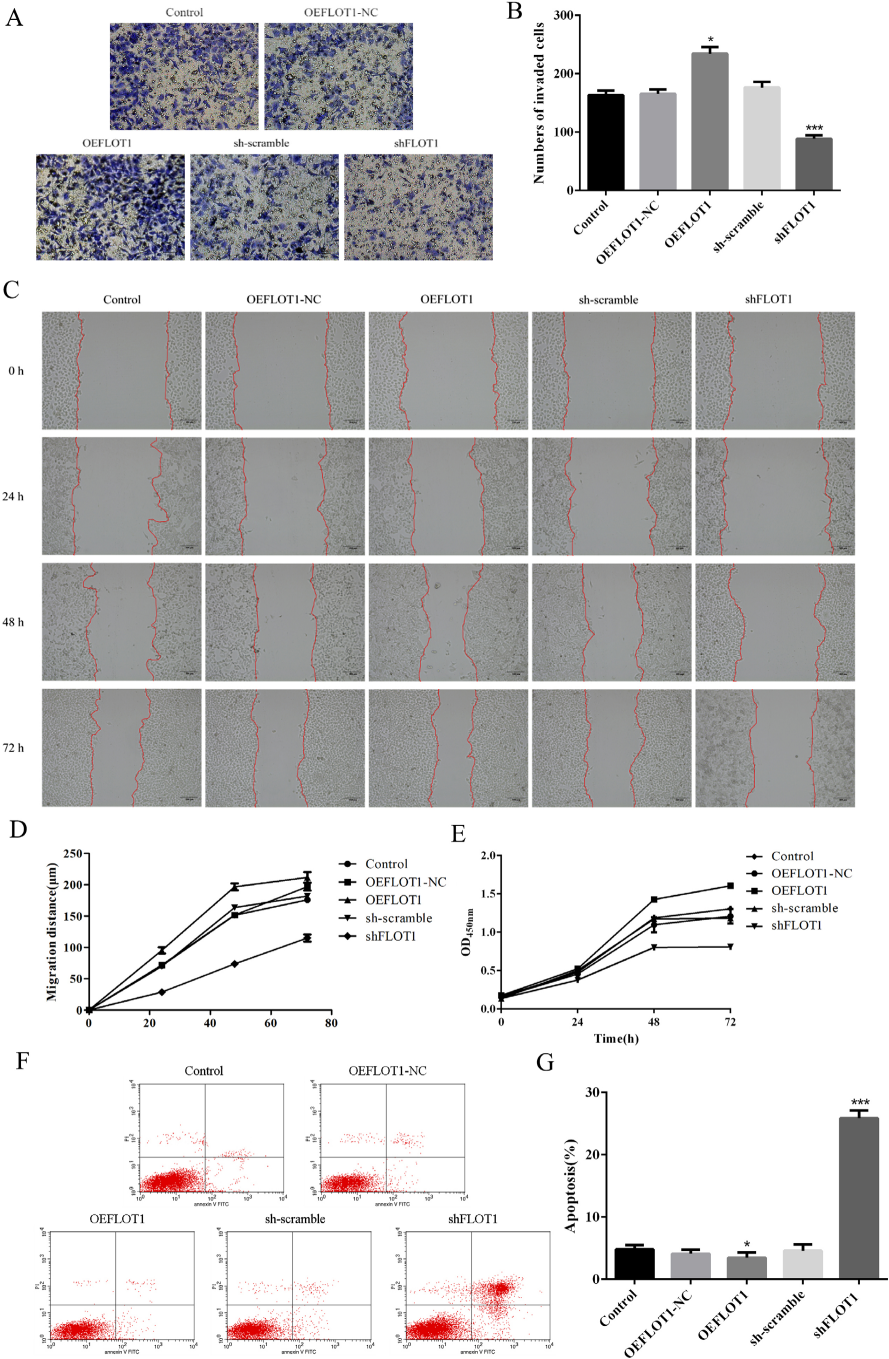

We apologize for these errors.

